# Association between serum folate levels and blood eosinophil counts in American adults with asthma: Results from NHANES 2011–2018

**DOI:** 10.3389/fimmu.2023.1134621

**Published:** 2023-02-22

**Authors:** Jun Wen, Changfen Wang, Mohan Giri, Shuliang Guo

**Affiliations:** ^1^ Department of Respiratory and Critical Care Medicine, The First Affiliated Hospital of Chongqing Medical University, Chongqing Medical University, Chongqing, China; ^2^ Department of Cardiology, The First Affiliated Hospital of Chongqing Medical University, Chongqing Medical University, Chongqing, China

**Keywords:** folate, eosinophil, asthma, national health and nutrition examination survey, machine learning

## Abstract

**Background:**

To date, many researches have investigated the correlation of folate and asthma occurrence. Nevertheless, few studies have discussed whether folate status is correlated with dis-ease severity, control or progression of asthma. So, we explored the correlation of serum folate and blood eosinophil counts in asthmatic adults to gain the role of folate in the control, progression, and treatment of asthma.

**Methods:**

Data were obtained from the 2011–2018 NHANES, in which serum folate, blood eosinophils, and other covariates were measured among 2332 asthmatic adults. The regression model, XGBoost algorithm model, and generalized linear model were used to explore the potential correlation. Moreover, we conducted stratified analyses to determine certain populations.

**Results:**

Among three models, the multivariate regression analysis demonstrated serum folate levels were negatively correlated with blood eosinophil counts among asthmatic adults with statistical significance. And we observed that blood eosinophil counts decreased by 0.20 (-0.34, -0.06)/uL for each additional unit of serum folate (nmol/L) after adjusting for confounders. Moreover, we used the XGBoost Algorithm model to identify the relative significance of chosen variables correlated with blood eosinophil counts and observed the linear relationship between serum folate levels and blood eosinophil counts by constructing the generalized linear model.

**Conclusions:**

Our study indicated that serum folate levels were inversely associated with blood eosinophil counts in asthmatic adult populations of America, which indicated serum folate might be correlated with the immune status of asthmatic adults in some way. We suggested that serum folate might affect the control, development, and treatment of asthma. Finally, we hope more people will recognize the role of folate in asthma.

## 1 Introduction

Asthma is a chronic airway disease with various etiologies (1, 2), affecting approximately 358 million people worldwide (3, 4). Epidemiological surveys have reported that about 250000 deaths are directly caused by asthma every year. And asthma was classified as the 23rd leading cause of premature mortality in 2016. With the development of society, the prevalence and socioeconomic burden of asthma are increasing year by year. According to reports, at present, the prevalence of asthma in adults is estimated to be 7.9% in the USA (5). Another study demonstrated that the cost of asthma treatment and mortality in the USA was estimated at $81 billion from 2008 to 2013 (6).

For many years, eosinophils in asthmatic inflammation have been recognized as an essential factor in the pathophysiology of asthma (7). Asthma is a heterogeneous disease comprising several endotypes and clinical phenotypes. Type 2 (T2) inflammation as key immune response in asthma pathobiology, resulting in the broad classification of T2-high and T2-low asthma. And eosinophils are also key inflammatory cells of ‘T2 high’ asthma (8). Quite a few studies reported a significant correlation between blood eosinophil counts and asthma (9–15). For example, eosinophilia has been confirmed to be a risk factor for future asthma exacerbations among asthmatic adults (16–18). And another study indicated increasing blood eosinophil counts might be correlated with rising mortality in patients with asthma (12). Besides, blood eosinophils are also used to guide and regulate treatment with corticosteroids, anti-IL antibodies in asthma, resulting in controlling asthma and reducing exacerbation frequency (19, 20). In short, eosinophils play a vital part in the occurrence, evolution, and therapy of asthma.

Asthma of underlying mechanisms maybe probably multifactorial, which may be affected by environmental, genetic, lifestyle factors, and so on, including dietary intake (21). A noticeable factor in a diet is folate. It takes part in synthesis of amino acid, purine, and pyrimidine, which is also a source of methyl donors for DNA methylation (22). Folate may contribute to the pathogenesis of asthma by affecting DNA methylation, further increasing or decreasing the expression of disease susceptibility genes (23, 24). Previous studies have reported, in animal models, folate plays a potential role in the pathogenesis of asthma (21, 24). In addition, some clinical researches reported that serum folate levels were correlated with the risk of asthma occurrence (21, 25–27).

At present, most studies have focused on exploring the relationship between folate and asthma occurrence. Nevertheless, to date, few researches have discussed whether folate status is correlated with disease severity or progression in asthmatic individuals; whether folate status affects asthma control is also unclear (24, 28). To gain insight into the role of folate in the control, progression, and treatment of asthma, we explored the correlation of serum folate concentration and blood eosinophil counts in asthmatic adults by using the data from the National Health and Nutrition Examination Survey (NHANES) of 2011–2018 cycles.

## 2 Materials and methods

### 2.1 Data source

The NHANES, which was conducted by the Centers for Disease Control and Prevention of America, collected information regarding the health and nutritional status of the U.S. population every 2 years. NHANES used a complex, stratified sampling design, which can select representative samples of non-institutionalized civilians. The NHANES database was approved by the NCHS Institutional Review Board in accordance with the revised Helsinki Declaration. The informed consent forms were completed before the data collection procedures and extensive health examinations.

### 2.2 Study population

**Figure 1:**
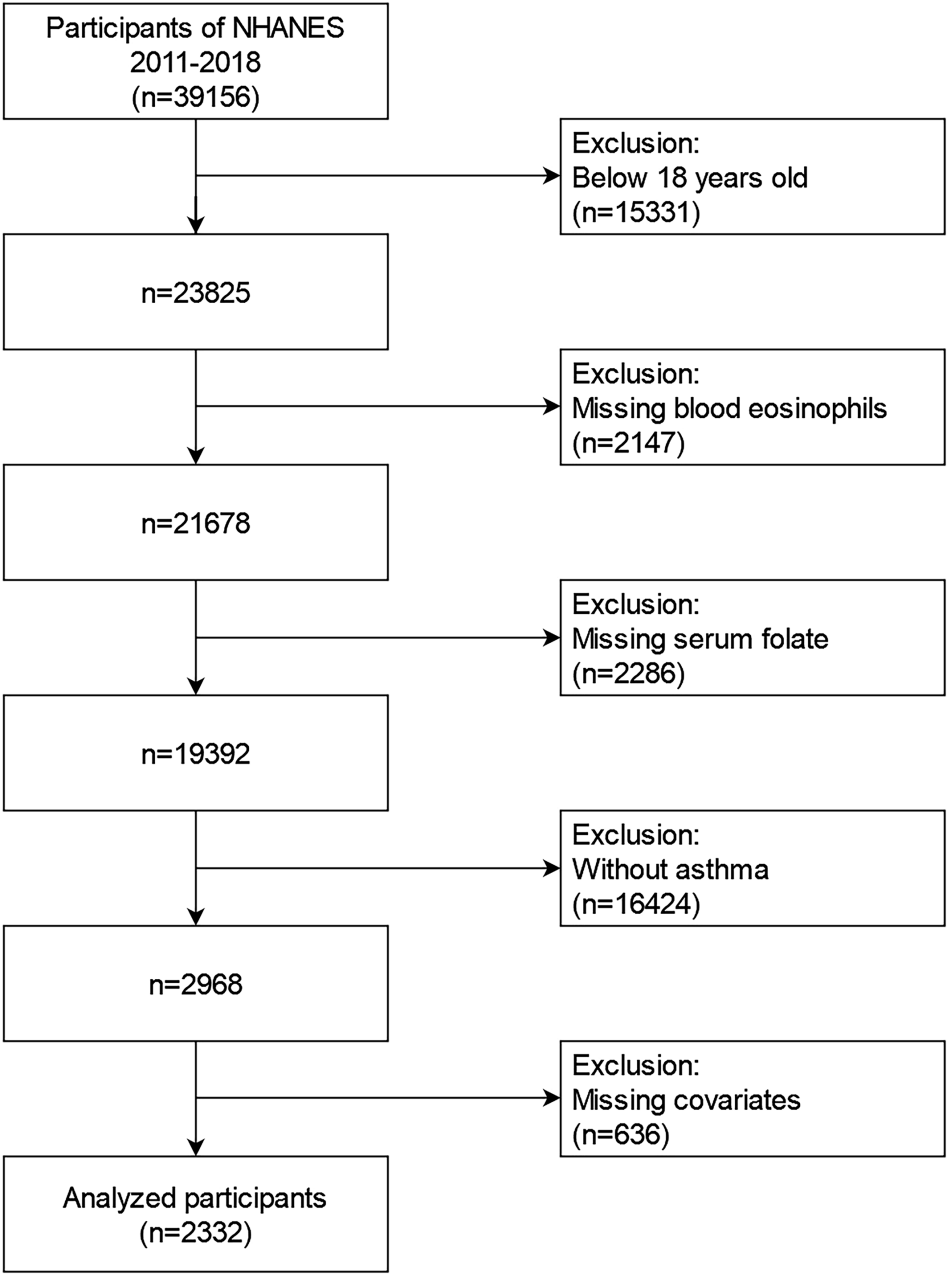
Flowchart for selecting analyzed participants.

2011-2018 NHANES data were involved into our research. These data contained demographic data, examination data, diet data, laboratory data, and questionnaire data for the second analysis. From 2011 to 2018, a total of 39156 samples were involved in the NHANES. We excluded individuals (1): aged<18 years old (n=15331) (2); missing blood eosinophils data(n=2147) (3); missing serum folate data (n=2286) (4); participants without asthma (n=16424) (5); missing data about covariates at least one of following (n=636): educational status, marital status, the ratio of family income to poverty (PIR), BMI, smoking status, intake of Vitamin A, Vitamin B12, Vitamin D or folate, housing, hypertension history, diabetes history, blood creatinine, and blood cotinine. Finally, a large national representative sample (n=2332) of American adults with asthma was enrolled in our research. The flow chart of the screening process is performed in **Figure 1**.

### 2.3 Measurement of serum folate

Folate in the blood contained five forms: 5-methyltetrahydrofolate, folic acid, tetrahydrofolate, 5-formyltetrahydrofolate, 5,10-methenyltetrahydrofolate. Finally, we use the concentration of total folate in serum to represent the overall level of five forms of folate in the blood. Total serum folate concentrations were measured by isotopedilution high performance liquid chromatography coupled to tandem mass spectrometry (LC-MS/MS). The assay is performed by combining a specimen (275 μL serum or whole blood hemolysate) with ammonium formate buffer and an internal standard mixture. Values below the lower limit of detection (LOD) for the various folate forms were imputed as LOD/Sqrt (2). Detailed instructions on specimen collection and processing can be found on the NHANES website.

### 2.4 Blood eosinophil count measurement

Blood differential counts were performed in NHANES 2011-2018 by using the Beckman Coulter HMX (Beckman Coulter, Fullerton, Calif), a quantitative and automated hematologic analyzer and leukocyte differential cell counter for *in vitro* diagnostic use in clinical laboratories. A detailed description of the laboratory methods can be found on the NHANES website.

### 2.5 Covariates and asthma assessment

Covariates were selected based on previous studies. Demographic data included gender, age (years old), race (Mexican American, other Hispanic, non-Hispanic white, non-Hispanic black, others), educational status (less than high school, high school, more than high school), poverty to income ratio (grouped by trisection: low, middle, high; high poverty to income ratio means richer), marital status (married, single, living with a partner). Secondly, we also contained examination data, diet data and personal life history data involving body mass index (kg/m2), smoking status (whether smoked at least 100 cigarettes in life), intake of Vitamin A, Vitamin B12, Vitamin D and folate (average intake from two 24-hours recall data on diet and supplements), housing (owned, rented, other), hypertension history (Yes, No), and diabetes history (Yes, No, Borderline). Finally, variables of laboratory data included blood basophil counts (/uL), blood creatinine (mol/L) and blood cotinine (ng/mL). The assessment of asthma was based on the information from the questionnaire section of the US National Health Interview Survey. In order to assess asthma, participants were asked, “Has a doctor or other health professional ever told you that you have asthma?” If the participant responds “yes,” he or she was regarded as asthma patient. More detailed explanations of all variables could be obtained from the NHANES database website (https://www.cdc.gov/nchs/nhanes/index.htm).

### 2.6 Statistical analysis

Based on the NHANES database guidelines, we conducted the statistical analyses of serum folate concentrations and blood eosinophil counts. Continuous and categorical variables were presented as the means ± SD and percentage, separately. Firstly, we converted blood eosinophil counts into four quartiles. We utilized the weighted chi-square to calculate the p-value of the basic characteristics of the analyzed individuals’ categorical variables and Kruskal Wallis rank sum test to calculate the p-value of continuous variables. Secondly, we constructed three types of weighted linear regression models which adjusted different variables to identify the association of serum folate concentrations and blood eosinophil counts (Model I, Model II, Model III). Thirdly, we utilized the machine learning of XGBoost algorithm model to discuss the relative significance of chosen variables on the effect of blood eosinophil counts. Next, we found the statistical difference between the serum folate concentrations and blood eosinophil counts. Therefore, we further conducted the stratified analysis to ascertain the stratified association of serum folate concentrations and blood eosinophil counts. Ultimately, according to the penalty spline method, we constructed a smooth curve by the generalized linear model to discuss the potential linear correlation of serum folate concentrations and blood eosinophil counts. All statistical analyses were conducted by R software (Version 4.2.0) using the R package. In our study, the p-value < 0.05 was indicated statistically significant.

## 3 Results

### 3.1 Baseline characteristics of the participants

The baseline characteristics, which were weighted distribution, were shown in **Table 1**, including demographic data, examination data, laboratory data, and questionnaire data of selected individuals from 2011-2018 NHANES survey. In our research, the mean age of selected individuals was 46.7 years old, and non-Hispanic White people were the main population. Then, we converted blood eosinophil counts into quartiles (Q1–Q4). The distribution of gender, age, hypertension history, blood creatinine, Vitamin A intake, Vitamin B12 intake, housing, diabetes history and blood basophil counts showed statistical differences (p values < 0.05), but the distribution of race, educational status, marital status, poverty to income ratio (PIR), housing, smoking status, diabetes history, Vitamin D intake, folate intake, blood cotinine and serum folate wasn’t statistically different (p value > 0.05).

**Table 1:** Weighted basic characteristics of the analyzed population based on the quartiles of blood eosinophils counts.

	Q1	Q2	Q3	Q4	P value
Gender (%)					0.001
Male	33.33	31.62	38.56	44.62	
Female	66.67	68.38	61.44	55.38	
Age, mean ± SD (years)	45.61 ± 1.86	43.07 ± 0.8	45.71 ± 0.88	46.62 ± 0.73	0.0101
Race/ethnicity (%)					0.0961
Mexican American	5.17	4.7	5.59	6.74	
Other Hispanic	5.8	5.22	5.8	6.24	
Non-Hispanic White	64.28	70.13	69.4	68.44	
Non-Hispanic Black	21.97	12	10.67	10.89	
Other Race	2.78	7.94	8.54	7.7	
Education (%)					0.1043
Less than high school	12.8	10.81	13.37	13.23	
High school	16.24	17.87	20.29	24.01	
More than high school	70.96	71.31	66.34	62.76	
Marital status (%)					0.5605
Married	42.69	52.01	48.82	52.12	
Single	52.7	40.43	42.39	40.28	
Living with a partner	4.62	7.56	8.79	7.6	
Poverty to income ratio, mean ± SD	2.52 ± 0.24	2.91 ± 0.09	2.75 ± 0.11	2.8 ± 0.1	0.1969
BMI, mean ± SD (kg/m2)	29.30 ± 1.06	29.69 ± 0.35	30.78 ± 0.48	31.49 ± 0.46	0.0033
Housing					0.3368
Owned	50.68	60.77	59.97	63.38	
Rented	45.09	37.13	36.72	35.34	
Other	4.23	2.1	3.31	1.29	
Smoked at least 100 cigarettes in life (%)					0.0539
Yes	50.71	43.48	45.25	52.96	
No	49.29	56.52	54.75	47.04	
Hypertension (%)					0.0022
Yes	37.16	29.93	36.73	42.05	
No	62.84	70.07	63.27	57.95	
Diabetes (%)					0.115
Yes	13.91	8.35	12.58	14.5	
No	82.81	89.49	85.04	82.91	
Borderline	3.28	2.16	2.38	2.58	
Vitamin A intake,mean ± SD (ug)	503.82 ± 58	621.97 ± 24.95	700.42 ± 27.6	645.37 ± 40.88	0.016
Vitamin B12 intake,mean ± SD (ug)	4.44 ± 0.6	4.74 ± 0.17	5.36 ± 0.24	5.27 ± 0.29	0.0397
Vitamin D intake,mean ± SD (ug)	3.35 ± 0.41	4.3 ± 0.25	4.92 ± 0.4	4.41 ± 0.23	0.0502
Folate intake,mean ± SD (ug)	364.53 ± 34.64	394.51 ± 14.14	417.58 ± 15.53	425.75 ± 17.03	0.2339
Blood creatinine,mean ± SD (umol/L)	73.56 ± 2.25	72.85 ± 0.93	77.3 ± 0.92	81.34 ± 2.36	<0.0001
Blood basophil counts,mean ± SD	31.92 ± 5.07	39.36 ± 2.53	56.98 ± 2.41	71.04 ± 2.73	<0.0001
Blood cotinine,mean ± SD (ng/mL)	82.93 ± 18.23	60.7 ± 7.38	56.77 ± 5.5	75.82 ± 7.44	0.0963
Serum folate,mean ± SD (nmol/L)	68.42 ± 25.37	44.01 ± 1.12	44 ± 1.5	42.9 ± 1.63	0.7209

Data are expressed as weighted means ± SD or proportions. Q1-Q4: Grouped by quartile according to the blood eosinophil counts. Our data included serum folate concentrations, blood eosinophil counts, demographic data, examination data, diet data, laboratory data, and questionnaire data for the second analysis.

### 3.2 The associations between the serum folate levels and blood eosinophil counts

We used the weighted linear regression models to discuss the correlation of blood folate and blood eosinophil counts in asthmatic adults (**Table 2**). According to the results, we observed that serum folate levels were inversely correlated with blood eosinophil counts in Model I, Model II and Model III, with statistical significance. In Model I, the blood eosinophil counts decreased by 0.17 (-0.29, -0.06)/uL for each additional unit of serum folate (nmol/L) (p for trend >0.05). In Model II, which adjusted for gender, age, and race, the blood eosinophil counts decreased by 0.18 (-0.30, -0.07)/uL for each additional unit of serum folate (nmol/L) (p for trend <0.05). In Model III, which adjusted for gender, age, race, educational status, marital status, PIR, BMI, smoked status, PIR, BMI, smoked, Vitamin A intake, Vitamin B12 intake, Vitamin D intake, folate intake, housing, hypertension history, diabetes history, blood basophil counts, blood creatinine, and blood cotinine, the blood eosinophil count decreased by 0.20 (-0.34, -0.06)/uL for each additional unit of serum folate (nmol/L) (p for trend >0.05). The above results indicated that serum folate might be correlated with the immune status of asthmatic adults in some way.

**Table 2:** Multivariate weighted linear regression model analysis elucidates the correlation between the serum folate and blood eosinophils counts.

	Model I	Model II	Model III
	β (95% CI) P value	β (95% CI) P value	β (95% CI) P value
Serum folate	-0.17 (-0.29, -0.06) 0.0052	-0.18 (-0.30, -0.07) 0.0027	-0.20 (-0.34, -0.06) 0.0076
Serum folate			
Q1	Reference	Reference	Reference
Q2	22.72 (-5.60, 51.04) 0.1212	21.37 (-6.53, 49.28) 0.1394	26.71 (0.89, 52.54) 0.0513
Q3	1.90 (-25.96, 29.76) 0.8940	0.65 (-27.43, 28.74) 0.9638	-2.06 (-28.32, 24.21) 0.8790
Q4	-11.96 (-29.41, 5.49) 0.1843	-17.30 (-37.29, 2.70) 0.0960	-17.50 (-41.69, 6.70) 0.1663
P for trend	-6.03 (-12.35, 0.29) 0.0661	0.0443	0.0639

Model I adjusts for none. Model II adjusts for gender, age and race/ethnicity. Model III adjusts for gender, age, race, educational status, marital status, PIR, BMI, smoked status, Vitamin A intake, Vitamin B12 intake, Vitamin D intake, folate intake, housing, hypertension history, diabetes history, blood basophil counts, blood creatinine, and blood cotinine. Q1-Q4: Grouped by quartile according to the serum folate.

### 3.3 Stratified associations between blood folate levels and blood eosinophil counts

To verify the stability of multivariate regression analysis results, we further analyzed stratified associations of the serum folate and blood eosinophil counts in different subgroups by sex, age, race, educational status, marital status, PIR, BMI, smoke, housing, hypertension history, and diabetes history (**Table 3**). According to stratified analysis results, it was possible that female, age <40, Non-Hispanic White people, more than high school, single status, high group of PIR, owned housing, BMI≥28, smoke≥100 cigarettes in life, without hypertension, and with diabetes had lower blood eosinophil counts, with increasing serum folate concentrations displaying a significant trend (p<0.05). Besides, we observed statistical differences in the interaction test. Variables of PIR, housing and serum folate may have interaction effects associated with blood eosinophil counts (p for interaction < 0.05).

**Table 3:** Stratified associations of association between the serum folate and blood eosinophils counts.

Serum folate (nmol/L)	N	β (95% CI) P value	P-interaction
Gender			0.9349
Male	914	-0.18 (-0.73, 0.36) 0.5116	
Female	1418	-0.21 (-0.34, -0.07) 0.0057	
Age			0.8137
<40	943	-0.24 (-0.33, -0.15) <0.0001	
40-60	725	-0.16 (-0.82, 0.49) 0.6291	
>=60	664	-0.12 (-0.53, 0.29) 0.5719	
Race/ethnicity			0.5747
Mexican American	213	-0.48 (-1.62, 0.65) 0.4115	
Other Hispanic	236	-0.99 (-2.09, 0.11) 0.0890	
Non-Hispanic White	1032	-0.19 (-0.33, -0.05) 0.0128	
Non-Hispanic Black	565	-0.35 (-0.89, 0.18) 0.2070	
Other Race	286	-0.12 (-0.86, 0.62) 0.7486	
Education			0.6454
Less than high school	424	-0.30 (-0.88, 0.29) 0.3260	
High school	487	0.07 (-0.54, 0.69) 0.8141	
More than high school	1421	-0.23 (-0.39, -0.07) 0.0100	
Marital status			0.3933
Married	1047	-0.53 (-1.11, 0.05) 0.0849	
Single	1092	-0.14 (-0.25, -0.03) 0.0165	
Living with a partner	193	0.14 (-1.45, 1.72) 0.8672	
Poverty to income ratio			0.0361
Low	776	0.18 (-0.41, 0.77) 0.5555	
Middle	775	0.21 (-0.10, 0.52) 0.1874	
High	781	-0.37 (-0.63, -0.11) 0.0081	
BMI			0.9325
<25	579	-0.24 (-1.02, 0.54) 0.5469	
25-28	424	-0.35 (-1.20, 0.51) 0.4331	
>=28	1329	-0.19 (-0.31, -0.07) 0.0046	
Smoked at least 100 cigarettes in life			0.7534
Yes	1101	-0.19 (-0.31, -0.08) 0.0024	
No	1231	-0.27 (-0.79, 0.24) 0.3075	
Hypertension			0.3167
Yes	958	0.04 (-0.52, 0.59) 0.9004	
No	1374	-0.25 (-0.41, -0.10) 0.0033	
Diabetes			0.7047
Yes	374	-0.18 (-0.34, -0.02) 0.0342	
No	1885	-0.23 (-0.53, 0.08) 0.1599	
Borderline	73	-0.68 (-1.81, 0.46) 0.2511	
Housing			0.0157
Owned	1237	-0.24 (-0.39, -0.08) 0.0062	
Rented	1039	0.07 (-0.42, 0.56) 0.7872	
Other	56	0.31 (-0.16, 0.79) 0.2077	

Above adjusts for gender, age, race, educational status, marital status, PIR, BMI, smoked status, Vitamin A intake, Vitamin B12 intake, Vitamin D intake, folate intake, housing, hypertension history, diabetes history, blood basophil counts, blood creatinine, and blood cotinine. In each case, the model was not adjusted for the stratification variable itself.

### 3.4 Utilizing the XGBoost algorithm model to discuss chosen variables’ relative significance

**Figure 2:**
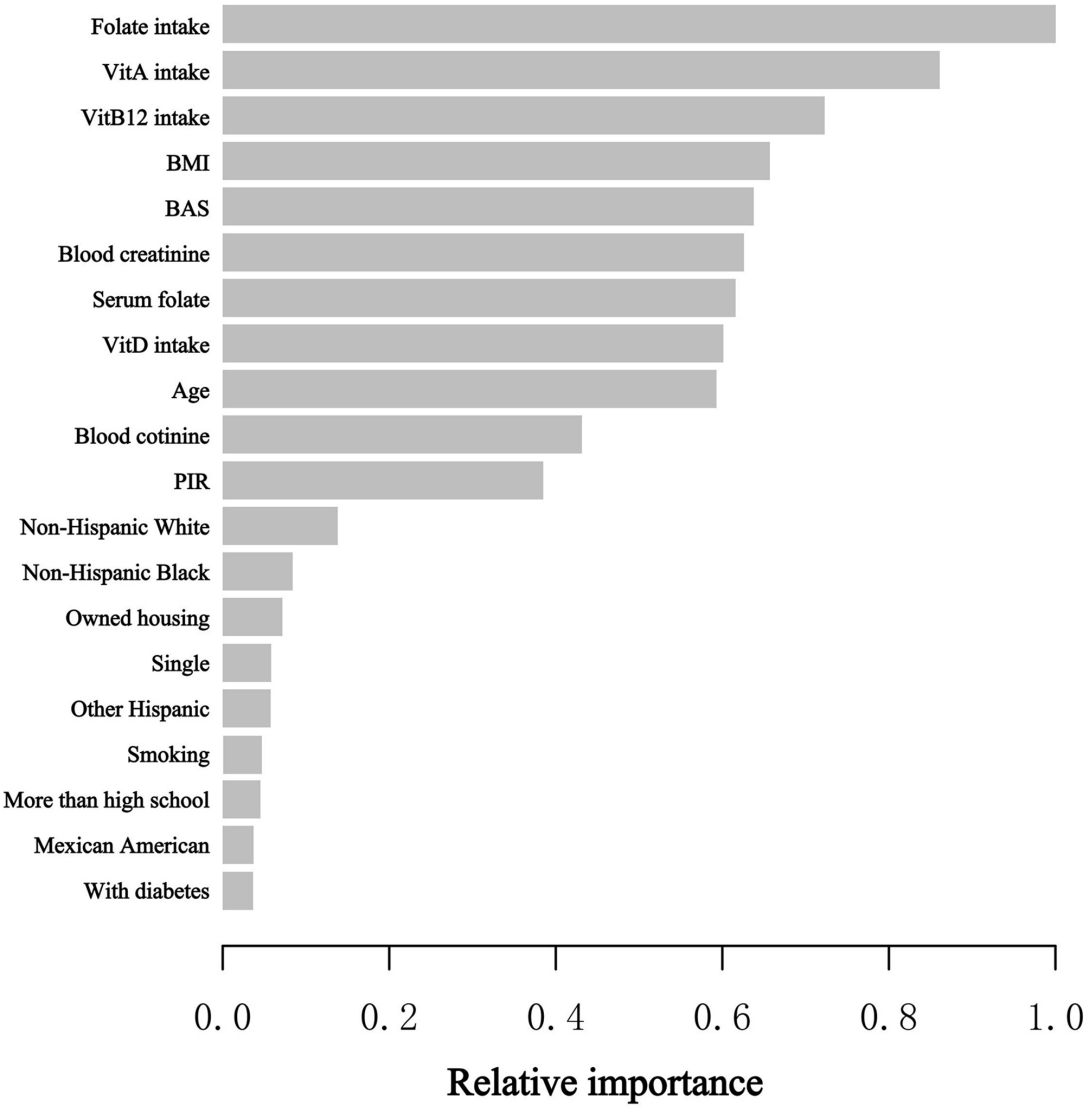
XGBoost model elucidates the relative significance of each variable on blood eosinophil counts and the corresponding variable significance score. The X-axis is the significance score, the relative number of a variable used to distribute the data; the Y-axis is the selected variables. PIR, poverty to income ratio; BAS, blood basophil counts; Vit, Vitamin.

At the stage of model development and validation, we utilized the XGBoost model of machine learning to identify the relative significance of chosen variables correlated with blood eosinophil counts. Variables contained sex, age, race, educational status, marital status, poverty to income ratio, BMI, smoking status, intake of Vitamin A, intake of Vitamin B12, intake of Vitamin D, intake of folate, housing, hypertension history, diabetes history, blood creatinine, blood cotinine, and serum folate. Based on the results of each variable contribution by the XGBoost model, we observed that folate intake, Vitamin A intake, Vitamin B12 intake, BMI, blood basophil counts, blood creatinine, serum folate, Vitamin D intake, age and blood cotinine, were the ten most important variables in the blood eosinophil counts (**Figure 2**). Ultimately, serum folate, as the relatively important variable, was further applied to constructing smooth curve models to verify the reliability of regression analyses results in our study.

### 3.5 Exploring dose-response relationships of serum folate levels with blood eosinophil counts by the generalized linear model

**Figure 3:**
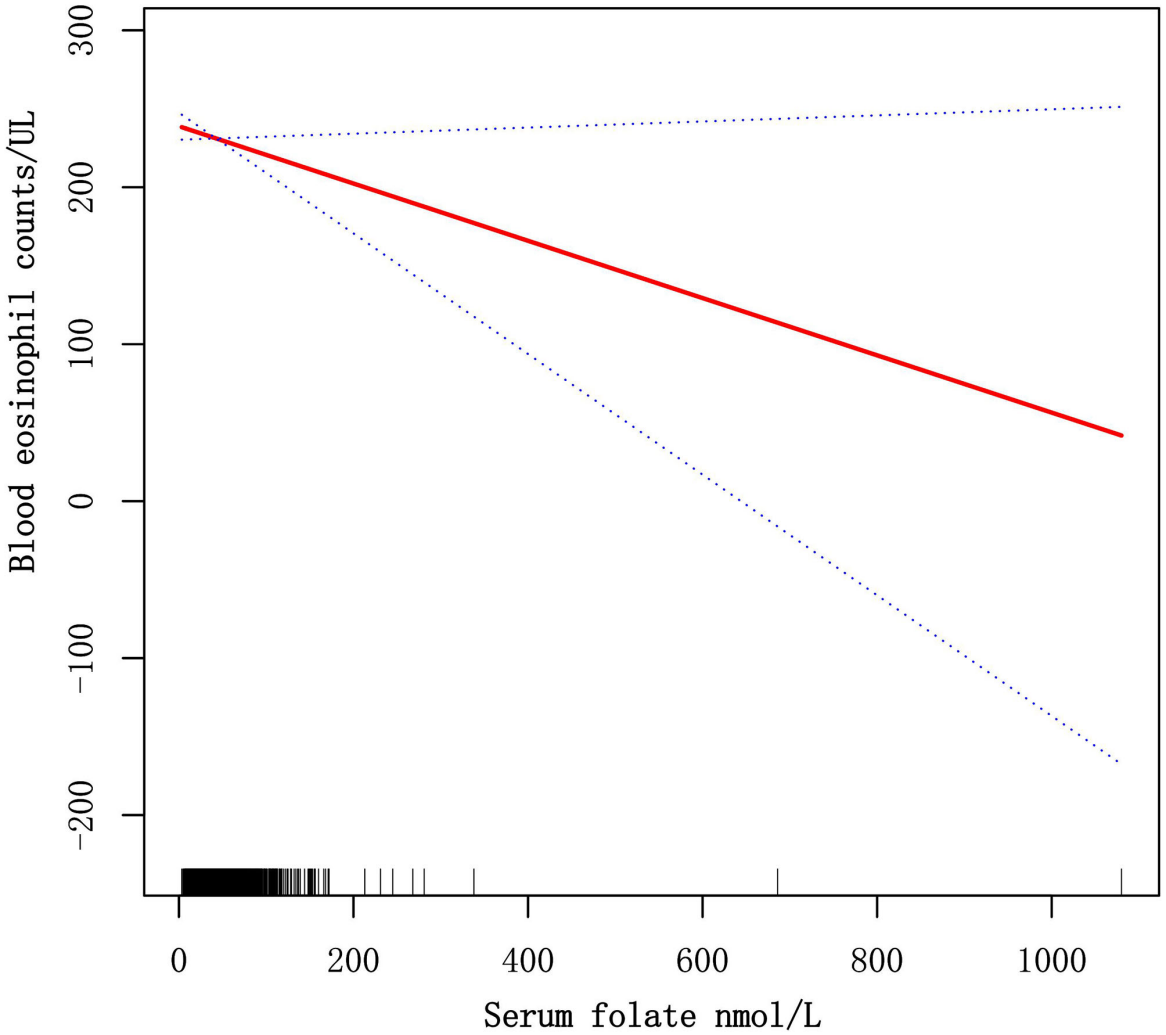
Dose-response relationships of serum folate levels with blood eosinophil counts. The solid red line represents the smooth fitting curve between serum folate levels with blood eosinophil counts, and the blue dotted line represents the 95% CIs of the fitting.

The GAM is greatly sensitive to identification of linear or nonlinear correlation. To verify the reliability of regression analyses results, we utilized GAM to discuss the linear or nonlinear correlation of the serum folate and blood eosinophil counts. Based on model 3 (**Figure 3**), we constructed a smooth fit curve to reflect the probable correlation. We observed the linear relationship between serum folate concentrations with blood eosinophil counts in asthmatic adults after adjusting all variables except for serum folate. All the above results indicated that serum folic acid levels were linearly and inversely associated with blood eosinophil counts.

## 4 Discussion

The prevalence of atopy has risen over recent decades, possibly because of changes in lifestyle and environmental factors. Recently, it was suggested that changes in the intake of certain nutrients might partly explain the asthma epidemic worldwide (29–31). Folic acid, a special nutritional component that has attracted much attention recently, is necessary for protein and DNA protein methylation, and for nucleotide synthesis (24). Folate could result in asthma risk by affecting DNA methylation and, further, genetic expression (23, 32). However, at present, there are few studies on the relationship between folate status with asthma severity or progression in asthmatic individuals. Thus, in our study, we used a largely representative sample of individuals who took part in the 2011-2018 of NHANES to explore the correlation of serum folate concentrations and blood eosinophil counts among asthmatic individuals.

To our knowledge, our investigation is the first research to discuss the correlation of serum folic acid levels and blood eosinophil counts in asthmatic adults and is also one of the most extensive cross-sectional studies. Therefore, we investigated the correlation of serum folic acid levels and blood eosinophil counts among 2332 asthmatic adults who took part in the NHANES survey from 2011 to 2018 in the USA. Three types of weighted multiple linear regression models were used to identify the association between the serum folate concentrations and blood eosinophil counts. Among three models, we all observed that serum folate levels were negatively correlated with blood eosinophil counts with statistical significance. Also, we observed that blood eosinophil counts decreased by 0.20 (-0.34, -0.06)/uL for each additional unit of serum folate (nmol/L) in the Model III, which adjusted for the demographic, laboratory, examination, dietary, and comorbidities data. Next, we constructed the machine learning of XGBoost model to identify the relative importance of chosen variables correlated with blood eosinophils, and we observed that folate intake, Vitamin A intake, Vitamin B12 intake, BMI, blood basophil counts, blood creatinine, serum folate, Vitamin D intake, age and blood cotinine were the ten most important variables in the blood eosinophil counts. Then, we observed that the linear relationship between serum folate concentrations and blood eosinophil counts through constructing the generalized linear model. The above results suggested that serum folate might be correlated with the immune status of asthmatic adults in some way.

DNA methylation, histone modifications and regulation of noncoding RNA, which are primary types of epigenetic regulatory mechanisms in humans, have been confirmed to be correlated with asthma, and might be regarded as biomarkers for asthma (33). DNA methylation, a crucial type of epigenetic regulation, is a mechanism underlying some gene-environment interactions of allergic diseases such as asthma. And DNA methylation plays a vital role in asthma through regulation of a substantial number of genes involved in allergic responses, indicating a strong correlation with the development of asthma and maintenance of inflammation *via* epigenetic control of IgE, eosinophils and fractional exhaled nitric oxide (FENO), among other mechanisms (34). Folic acid, a water-soluble vitamin, is called vitamin B9, too. It plays a critical role in purine, pyrimidine and amino acid of synthesis, which is also a source of methyl donors for DNA methylation. In a mouse model, one animal experiment demonstrated that a diet rich in folate enhanced allergic humoral responses and lung inflammation. However, in contrast, some studies reported that lower folate levels were correlated with various inflammation-mediated diseases such as rheumatoid arthritis, inflammatory bowel disease (35, 36), and cardiovascular disease (37–39). Thus, it is possible that folic acid may alleviate, rather than promote, allergic diseases.

Several clinical studies have reported the association between folate concentrations and asthma. In Denmark, research including 6784 adults observed that obvious correlations between folic acid of deficiency and physician-diagnosed asthma (40). One case-control study including 1030 adults in the UK observed that intake of folate was negatively correlated with physician-diagnosed asthma (41). But another case-control study including 1469 adults in Australia observed that no correlation between intake of folate and physician-diagnosed asthma (42). And a case-control study involving 754 Peruvian children observed that serum folic acid concentrations were negatively associated with asthma (21). In addition, a Korean study involving 6615 individuals aged >10 years found that serum folic acid concentrations were negatively correlated with physician-diagnosed asthma (26). In contrast, a case-control study in Egypt, which involved 180 adults, found no significant association between serum folate levels and asthma (43). Likewise, in another cross-sectional study including 8083 American participants aged 2–85 years, no significant correlation between serum folate levels with physician-diagnosed asthma was observed (25). Besides, in a prospective cohort study, which mainly included black, urban children and adolescents with asthma, researchers observed that there are significant associations between serum folic acid and FENO and total IgE levels (28). Previous studies demonstrated that increasing folate could serve as a protective factor in certain populations against allergic inflammatory outcomes such as wheeze, atopy, and high total IgE. For example, a cross-sectional study in the USA, which involved 8083 participants aged 2 to 85 years old, observed that serum folic acid concentrations were negatively correlated with wheeze, total IgE, and atopy (25). Likewise, an Egyptian study involving 120 asthmatic adults demonstrated that serum folate was inversely correlated with total IgE, too (43). And lower serum folate concentrations were correlated with a higher risk of uncontrolled asthma in a study of 412 asthmatic children (21). However, above most studies, which explored the correlation of serum folate and asthma, didn’t include accommodation and eating data such as intake of Vitamin A, Vitamin B12, Vitamin D, folate and so on, meanwhile, had small sample populations. In addition, these studies didn’t carry out stratified analyses in various populations to confirm the stability and reliability of analyzed results. Our results were similar to those of the above studies, which demonstrated that serum folate levels were inversely associated with blood eosinophil counts in asthmatic adults. What’s more, we observed that the association between serum folic acid concentrations and blood eosinophil counts had population differences among American asthmatic adults, too. Based on the stratified analysis, we determined possibly protective groups with higher folate levels, including female, age <40, Non-Hispanic White people, more than high school, single status, high group of PIR, owned housing, BMI≥28, smoke≥100 cigarettes in life, without hypertension, which had lower blood eosinophil counts. In a word, serum folate levels may potentially affect the control and progression of asthma through immunomodulation and modulation of inflammation.

Compared with previous investigations, our study has several advantages. Firstly, our research offers a nationally representative, relatively large sample of asthmatic adults, which includes information on a few potential confounders. Second, considering that confounders may affect the results, we identify the possibly protective population with higher folate levels through stratified analysis. Then, we use the machine learning of XGBoost Algorithm model to identify, among all selected variables, serum folate affects blood eosinophil counts most. Finally, we observed the linear relationship between serum folate levels and blood eosinophil counts by constructing the generalized linear model.

Nonetheless, we still acknowledge several limitations in our study. Though our study is national broad, most of the data is based on the American population. And dietary model may be different in different countries on account of unbalance in national development. Secondly, due to the cross-sectional study design limits, we can’t conclude the causal relationship between serum folate and blood eosinophil counts. Finally, we may not take certain potential confounders into consideration. Moreover, in future, information such as lung function, airflow obstruction, atopy and so on will be included into further studies. And more prospective researches will be conducted to get insight into the potential role of folate in the control, progression and treatment of asthma, and to determine potential mechanisms of action.

## 5 Conclusion

Our study observed that serum folate levels were inversely associated with blood eosinophil counts in asthmatic adult populations of America, which indicated serum folate might be correlated with the immune status of asthmatic adults in some way. We suggested that serum folate might affect the control, development, and treatment of asthma. Finally, we hope more people will recognize the role of folate in asthma.

## Data availability statement

The datasets presented in this study can be found in online repositories. The names of the repository/repositories and accession number(s) can be found below: http://www.cdc.gov/nchs/nhanes/.

## Ethics statement

The studies involving human participants were reviewed and approved by the National Centers for Disease Control (CDC) and Prevention National Health Statistics Center. The patients/participants provided their written informed consent to participate in this study.

## Author contributions

JW conducted the study design, data extraction, statistical analysis, drafted the manuscript, and revision of the paper. MG conducted data extraction, statistical analysis, and revision of the paper. CW conducted the study design, statistical analysis, drafted the manuscript, and revision of the paper. SG took part in the study design and revision of the paper. All authors contributed to the article and approved the submitted version.
